# Multi-domain improves classification in out-of-distribution and data-limited scenarios for medical image analysis

**DOI:** 10.1038/s41598-024-73561-y

**Published:** 2024-10-18

**Authors:** Ece Ozkan, Xavier Boix

**Affiliations:** 1 Department of Brain and Cognitive Sciences, MIT, Cambridge, 02139 USA; 2 Department of Computer Science, ETH Zurich, 8092 Zurich, Switzerland; 3 Fujitsu Research of America, Inc., Sunnyvale, 94085 USA

**Keywords:** Multi-domain, Generalizability, Out-of-distribution, Medical image analysis, Medical research, Biomedical engineering

## Abstract

Current machine learning methods for medical image analysis primarily focus on developing models tailored for their specific tasks, utilizing data within their target domain. These specialized models tend to be data-hungry and often exhibit limitations in generalizing to out-of-distribution samples. In this work, we show that employing models that incorporate multiple domains instead of specialized ones significantly alleviates the limitations observed in specialized models. We refer to this approach as *multi-domain model* and compare its performance to that of specialized models. For this, we introduce the incorporation of diverse medical image domains, including different imaging modalities like X-ray, MRI, CT, and ultrasound images, as well as various viewpoints such as axial, coronal, and sagittal views. Our findings underscore the superior generalization capabilities of multi-domain models, particularly in scenarios characterized by limited data availability and out-of-distribution, frequently encountered in healthcare applications. The integration of diverse data allows multi-domain models to utilize information across domains, enhancing the overall outcomes substantially. To illustrate, for organ recognition, multi-domain model can enhance accuracy by up to 8% compared to conventional specialized models.

## Introduction

In medical image analysis, existing machine learning approaches propose models to address wide range of problems^1–3^, which have been tailored for their designated applications and typically utilize data from a single target domain. This approach leads to data-intensive specialized models and show limited generalization capabilities. Proposed works in medical image analysis falls short of fully leveraging the diverse medical image data available. Various imaging modalities, such as X-rays, magnetic resonance imaging (MRI), computed tomography (CT), ultrasound (US), and positron emission tomography (PET), provide unique perspectives into different aspects of anatomy and pathology. X-rays excel in revealing bone structures and detecting fractures, while MRI scans provide detailed images of soft tissues like the brain, muscles, and organs. CT scans offer cross-sectional views, helping to identify internal injuries and complex conditions. US images are non-invasive and excel in real-time imaging, often used for monitoring pregnancies and examining internal organs, whereas PET provides metabolic information, aiding in cancer detection and localization. The combination of these imaging modalities is common in clinical practice and enhances diagnostic accuracy by providing complementary information that might not be evident in a single imaging method. Furthermore, in medical decision-making, clinicians often consider diverse viewpoints, since certain anomalies may be more apparent from one angle than another, ensuring a comprehensive understanding of the patient’s condition and facilitating accurate diagnoses and effective treatment strategies.

Our work seeks to address a pivotal question: Can the integration of diverse image domains, such as medical imaging modalities or viewpoints, improve the generalization capabilities of models for a specific task in medical image analysis? To answer this question, the main contributions are as follows:

- We introduce a *multi-domain* model, with diverse medical image data domains, such as imaging modalities, like X-ray, MRI, CT, and US images, or various viewpoints, such as axial, coronal, and sagittal views, This model uses the data from different imaging domains to train for a specific task with an off-the-shelf architecture. For comparison, we also train conventional specialized models for the same task using data exclusively from each individual domain.
- We evaluate the performance of specialized models in comparison to multi-domain model using three publicly available datasets, such as PolyMNIST^4^, MedMNIST^5^ and ImageCLEFmedical^6^. We compare their accuracy for out-of-distribution (OOD) and data-limited scenarios, common in healthcare applications, showing that the integration of diverse data allows multi-domain models to enhance the overall outcomes in comparison to specialized models.

To illustrate this idea, consider the following question: Can a neural network trained on instances of a medical condition as observed through CT, PET, and X-ray images, provide accurate predictions when presented with MRI images, even in cases where this condition has been encountered infrequently in the training set from MRI images? This work represents the first instance where multi-domain data is assessed for a single task, different from multi-task setups. It provides insights into the respective strengths and drawbacks of these models, showing the potential of diverse data domains in medical image analysis applications.

### Related work

Recent years have witnessed a rise of foundation models, particularly in fields like natural language processing and computer vision. These models combine data from various domains and demonstrate exceptional generalization capabilities beyond their primary training tasks. In the sphere of LLMs, noteworthy examples include^7–17^, where some of these models utilized clinical notes and electronic health records in their development. Additionally, large vision models for healthcare have emerged, driving significant advancements across diverse applications^18^. For instance,^19–26^ utilize self-supervised learning for different tasks using single imaging modality such as CT, MRI or X-rays. Efforts for multi-organ segmentation tasks also exist, such as^27–33^. These models have not only expanded in terms of their number of parameters and data handling capacities but have also consistently demonstrated remarkable performance once pre-trained. There have been some efforts to develop medical vision foundation models using diverse data, however, their widespread adoption remains limited.

When aiming to enhance generalization capabilities, an alternative approach to consider is multi-task learning^34^. Here, the goal is to improve the performance of a model while solving multiple related tasks simultaneously. The idea is that learning from multiple tasks can help the model capture shared patterns and representations, leading to better performance on each individual task. In contrast, in our work we focus on training with data from different domains without necessarily involving multiple tasks. As a practical example, our setup can be trained to identify abnormalities across CT, MRI, US and PET images. In contrast, multi-task learning focuses on detecting abnormalities while learning another related task within a single image domain, such as using MRI scans.

Several studies have explored the role of multi-modality approaches in healthcare contexts^22,24,25,35,36^. However, these methods predominantly focus on integrating text with a single imaging modality, rather than incorporating data from various image domains. Furthermore, our approach differs by requiring only a single input from any one modality, rather than needing multiple simultaneous inputs. This flexibility is crucial in clinical settings where some modalities may be unavailable. Unlike multi-modal models that may struggle with missing data, our multi-domain model leverages shared information across modalities to maintain robust performance even when only one modality is present. Closest work to ours is BenchMD^37^, where they combined 19 publicly available datasets for 7 medical modalities, including 1D sensor data, 2D images, and 3D volumetric scans. In the case of 2D images, BenchMD combined data from diverse sources, including chest X-rays (CXR), mammograms, dermoscopic, and fundus images. They utilized widely-cited and large dataset as the primary source for each imaging modality, conducting evaluations of distribution shifts on a separate test set. For this they train a single unified model across all the modalities or domains included in the benchmark. However, each dataset is associated with distinct classes that are not shared across modalities, which is different from our approach, where the same classes are shared across multiple modalities.

Our method is most related to works on multi-domain networks^38–41^, which focus on training a single network to handle image classification tasks across diverse domains. The primary objective is to develop a single network capable of compactly representing all domains with minimal task-specific parameters. These models introduce various architectures to incorporate diverse data domains into the model parameterization and assess their models’ generalizability across different natural image datasets. In the healthcare domain,^42^ proposed a self-supervised representation learning method that integrates multiple domains into the learned representations. However, our work differs from these methods by leveraging multi-domain data to train for a specific task using an off-the-shelf architecture.

In Table 1, we present a summary of our work, comparing it to related work based on their input, output, and the data needed for training and testing.

**Table 1:** Summary of our work, the multi-domain model, compared to related work of specialized, multi-task and multi-modal models based on their input, output, and the data needed for training and testing.

	Input [# Images]	Output [# Tasks]	Dataset [# Domains]	Input instance [# Domains]
Specialized	Single	Single	Single	Single
Multi-task	Single	Multiple	Single	Single
Multi-modal	Multiple	Single	Multiple	Multiple
Multi-domain	Single	Single	Multiple	Single

## Methods

The level of generalization that multi-domain models can achieve in scenarios involving out-of-distribution and limited data remains uncertain based on prior research involving large scale models applied to medical image analysis^9,37,43–45^. We aim to explore the potential for shared information across different data domains, such as imaging modalities or viewpoints. To achieve this, we will first introduce the datasets employed in this study and then outline the methods used to generate data diversity within these datasets, thereby allowing us to analyse the impact of diverse data domains on generalizability.

### Datasets

Most existing datasets from various data domains are tailored to their specialized applications and these datasets lack commonalities that would allow for evaluating potential knowledge transfer. Thus, we present our results on the following datasets, which do not suffer from these challenges.

#### PolyMNIST

**Fig. 1:**
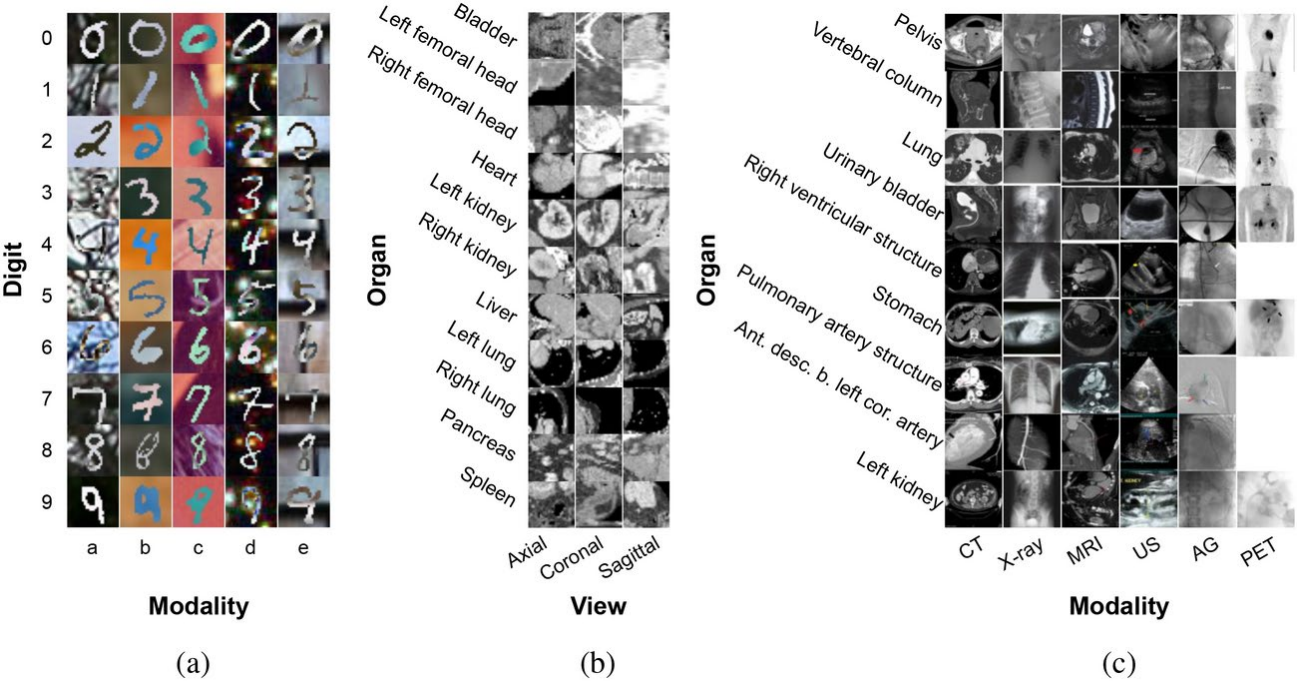
We employ (**a**) PolyMNIST, a dataset for digit classification with data from different modalities, (**b**) MedMNIST for the classification of organs from different views of CT image slices, and (**c**) ImageCLEFmedical for organ classification using data from different imaging modalities. For each dataset, the rows represent the classes, and the columns represent the domains.

We start with the multi-modal benchmark PolyMNIST^4^ to understand behaviours for different ablations. The PolyMNIST dataset consists of sets of ten MNIST digits where each set includes five images with the same digit label but different backgrounds and different styles of hand writing. Here, we adopt the terminology used by authors in^4^, where they refer to each background as a “modality” to capture source specific information. Thus, for our experiments, each digit represents the shared information across modalities and different background images represent modality-specific information. In total we used for each digit and modality combination 1000 samples of training and validation examples (50000 images in total for ten digits and five modalities) and 891 samples of test examples (44550 images in total) from the original train and test split of the dataset. Our objective is to perform multi-class classification of ten digits across five different modalities, as shown in Fig. 1(a).

#### MedMNIST

**Fig. 2:**
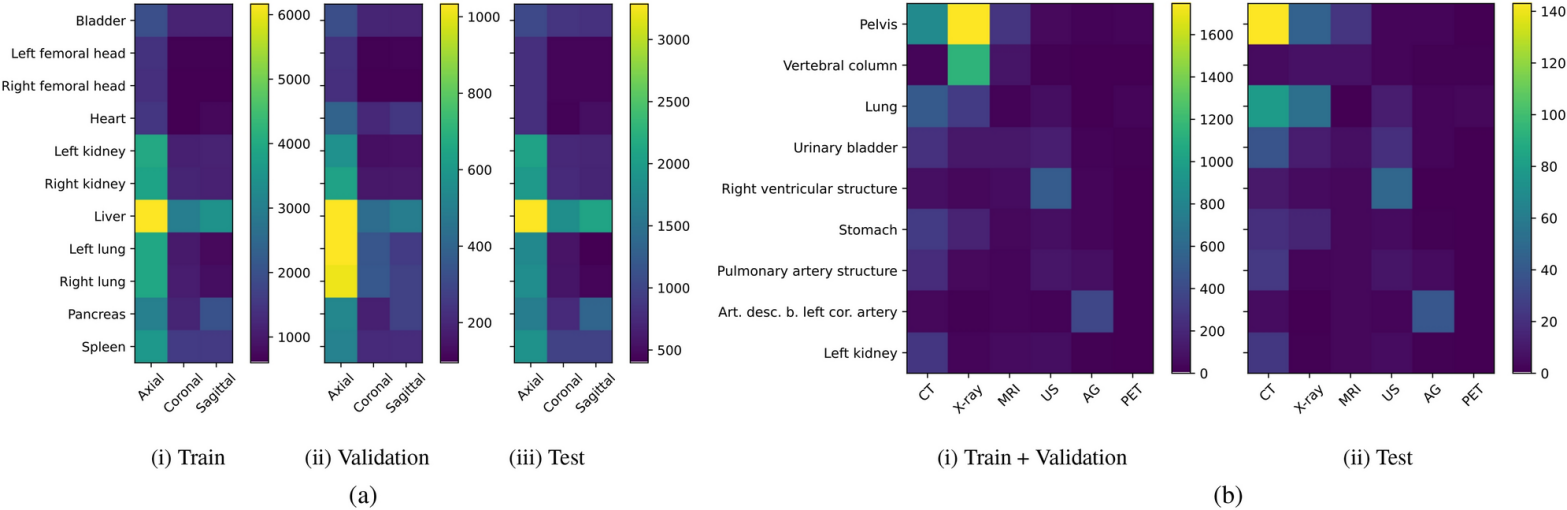
Number of images for medical datasets: (**a**) MedMNIST showing the distribution across (i) training, (ii) validation, and (iii) test sets. (**b**) ImageCLEFmedical detailing the distribution in (i) combined training and validation set, and (ii) test set.

We use MedMNIST v2^5^ benchmark to explore generalization across viewpoints. MedMNIST v2 is a large-scale MNIST-like dataset collection of standardized biomedical images, including datasets of 2D and 3D data. Among these, we use Organ{A,C,S}MNIST subset, which are based on CT images from *axial, coronal* and *sagittal* views. The visible organs within this data include *bladder, left femoral head, right femoral head, heart, left kidney, right kidney, liver, left lung, right lung, pancreas* and *spleen*. We used the original data split, with 61521 training 11335 validation and 34875 test samples. The goal is to perform multi-class classification of 11 organs from axial, coronal and sagittal views. Examples of organ and view combinations are shown in Fig. 1(b). Number of samples for each combination are shown in Fig. 2(a) and Table A.1, with (i) representing the training, (ii) validation, and (iii) test set.

We would like to point out that while multi-view involves different perspectives of the same 3D volume, we consider it a form of multi-domain in this work because each view provides unique and complementary information. Although these views come from the same source, the variation between them can introduce challenges similar to those found in distinct domains, making multi-view a relevant subset of the multi-domain concept in our analysis.

#### ImageCLEFmedical

We use ImageCLEFmedical Caption challenge^6^ dataset, a subset of the extended Radiology Objects in COntext (ROCO) dataset^46^. This dataset is derived from biomedical articles within the PMC OpenAccess subset, a comprehensive collection of figures sourced from open access biomedical journal articles (PubMed Central), along with radiology images extracted from original medical cases. In both training and validation data, each image is paired with Unified Medical Language System(UMLS) 2020 AB concepts^47^. These concepts represent UMLS terms, recognized as as CUIs (Concept Unique Identifiers) and extracted from the accompanying image captions. For instance, if the image caption contains terms such as “plain x-ray” or “pelvis”, these concepts would be denoted for that image by the CUIs C1306645 and C0030797. Note that the ImageCLEFmedical Caption challenge comprises two subtasks: concept detection and concept prediction. Our experiments utilize the concept detection data within this dataset.

There are more than 8000 concepts in the dataset, each with varying frequencies of occurrence. To enhance control and comprehension of generalization, we opted to work with a subset of images and concepts. Our analysis focused on the 100 most frequently employed CUIs. Filtering was then applied to images based on the semantic types of their associated concepts, specifically targeting concepts related to “Diagnostic procedure” for imaging modality identification, and “Body Part, Organ, or Organ Component” for presence of specific organs in the images. From the filtered list of CUIs, we selected a subset of organs for further analysis. This subset comprised nine distinct body organs, namely *pelvis, vertebral column, lung, urinary bladder, right ventricular structure, stomach, pulmonary artery structure, anterior descending branch of the left coronary artery,* and *left kidney*. As for imaging modalities, we considered *CT, X-ray, MRI, US, angiogram (AG),* and *PET* images. It’s important to note that not all body parts are captured through all imaging modalities. Since the test images in the original dataset do not come with their concepts, we employed the train split from the original challenge dataset for training and validation, while the validation split was repurposed as the test set. This resulted in a dataset of 8433 images for training and validation, with an additional 688 images reserved for testing. Our task entails multi-class classification of the nine body organs across six distinct imaging modalities. Examples of body organ and modality combinations are shown in Fig. 1(c). Furthermore, the number of samples for each combination are visualized in Fig. 2(b) and Table A.2, where (i) illustrates the combined train and validation, and (ii) the test set.

### Dataset specific classes and domains

In our experiments, we focus on classifying different digits for PolyMNIST or organs for the medical datasets across different data domains. The datasets can be visualized as a grid structure illustrated in Fig. 1 and each square within this grid represents a unique class/domain combination. These combinations consist of digit/modality combination for PolyMNIST, organ/view combination for MedMNIST and organ/modality combination for ImageCLEFmedical. Each row corresponds to a class, whether it be a digit or an organ, while each column signifies a specific data domain, such as a view or a modality. Each cell within the grid includes all the images from a particular combination of class and domain.

### Generating train and validation splits for data diversity

We create different data subsets characterized by differing aspects and levels of data diversity. We base the generation of these partitions on two key factors: data availability and the level of OOD, which we will explain in more detail below. During the testing phase, we refrain from any additional data processing and exclusively employ the predefined test splits as provided within each dataset.

#### Amount of data

**Fig. 3:**
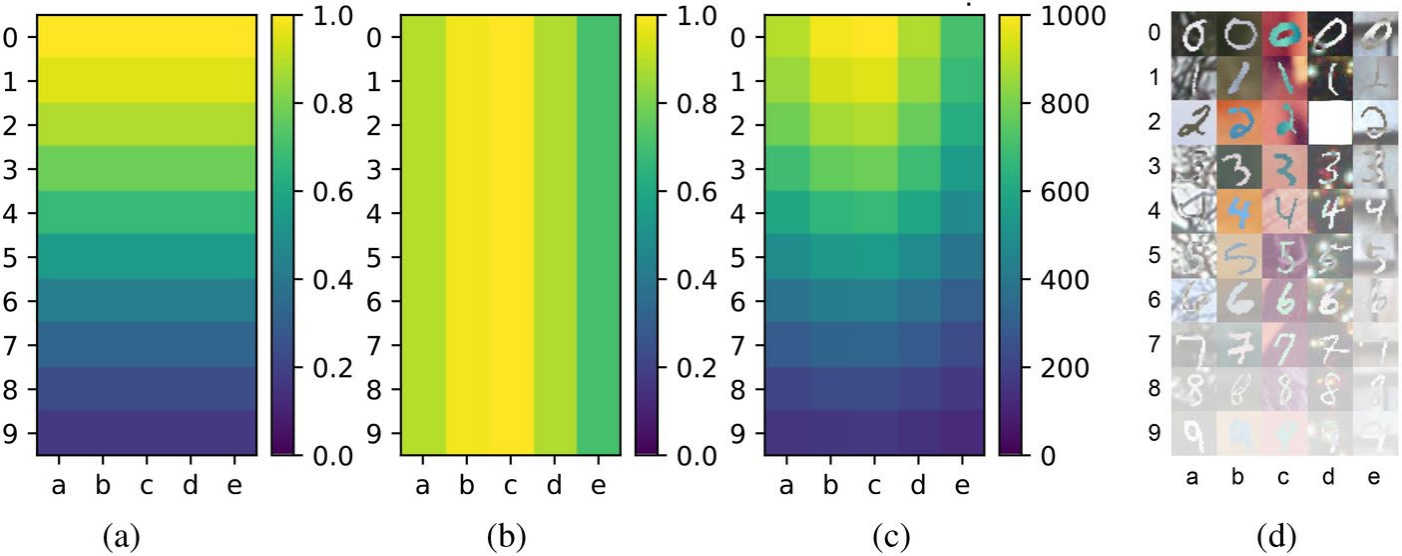
Generating data diversity for PolyMNIST: (**a**) normalized digit distribution using $\mu _\text {digit}=0$ and $\sigma _\text {digit}=5$, (**b**) normalized modality distribution with $\mu _\text {modality}=2$ and $\sigma _\text {modality}=3$, (**c**) number of samples from the resulting distribution for each digit/modality combination, (**d**) example of data distribution and out-of-distribution scenario with a level of 100% for digit *2* and modality *d*.

For PolyMNIST, we start by constructing train and validation splits with different data distributions. For each of the digit and modality combination in the set, we have access to 1000 samples. To introduce diversity to data distribution, we implemented diverse probability distributions for digit and modality combinations, as follows. We use normal distribution for digit and modality distributions with the following parameters: the mean of the digit distribution, denoted as $\mu _\text {digit}$ is set to 0, and the standard deviation, $\sigma _\text {digit}$, takes values from the set $\{3,5,9,17\}$. For the modality distribution, the mean, $\mu _\text {modality}$, is chosen from $\{0,2\}$ , while the standard deviation, $\sigma _\text {modality}$, is selected from $\{1,3,5\}$. We normalized these distributions, ensuring that all values are within the range of 0 to 1. We then multiply the digit and modality distribution functions and rescale the product by a factor of 1000, hereby guaranteeing that each distinct modality and digit combination has a sample count between 0 and 1000. In Fig. 3(a–c), we present a graphical representation of this process, employing the specific parameter values of $\mu _\text {digit}=0$, $\sigma _\text {digit}=5$, $\mu _\text {modality}=2$, and $\sigma _\text {modality}=3$. The combination of different mean and standard deviation parameters for digit and modality distribution yields a total of 24 distinct distributions to use, as shown in Fig. A.1.

Note that the datasets for both MedMNIST and ImageCLEFmedical inherently exhibit diversity, since they have varying sample counts for each organ across different views in MedMNIST and across different modalities in ImageCLEFmedical, as shown in Fig. 2. Thus, we employ a range of sampling percentages to create distinct training and validation subsets for these datasets. We use their provided distributions and sample training and validation subsets accordingly. The sampling percentages include $\{5,10,25,35,50,75,100\}$%, where 100% indicates the utilization of the entire available training and validation data, while 50% implies that only 50% of the data is incorporated. For instance, when employing a 100% sampling rate for MedMNIST, the multi-domain model utilized the complete training split consisting of 61521 images, 11335 images from the validation split, and 34875 images from the test split. At a 50% sampling percentage, the model then utilized 50% of the total available training and validation samples, resulting in 30760 samples from the training set, 5668 samples from the validation set, and retained the full 34875 test samples for evaluation. As a result, this sampling approach ensures consistent ratios of training and validation samples across different combinations, albeit with varying sample sizes.

#### Out-of-distribution (OOD) level

To evaluate the OOD performance of both specialized and multi-domain models across various datasets, we introduce *OOD levels*, as follows: for each class and data domain combination, we systematically exclude a subset of instances from both the training and validation sets. Subsequently, we repeat the training and validation procedures for each of the combination. We quantify OOD levels using percentages, specifically $\{0,25,50,75,85,95,100\}\%$, where 100% signifies that the specific combination is entirely absent from both the training and validation sets, whereas, 0% indicates that the training and validation datasets contain the complete set of samples for the given combination. As a result, for each experiment, the specific combination becomes either never or less frequently observed. For a fair representation of each combination, we ensure that every combination occurs exactly once, that is, each row and column features only a single cell representing a specific combination.

We present an illustrative example of diverse data strategies for PolyMNIST in Fig. 3(d). Here, opacity is used to represent the quantity of data, with less opacity indicating a larger amount of data. Specifically, we employed distribution parameters $\mu _\text {digit}=0$, $\sigma _\text {digit}=5$, $\mu _\text {modality}=2$, and $\sigma _\text {modality}=3$ to define the data distribution, as in Fig. 3(c). Furthermore, it also showcases the *OOD scenario* with a 100% OOD level for digit *2* and modality *d*, meaning the model will never see the digit of *2* from the modality *d*.

### Training and testing procedure

**Fig. 4:**
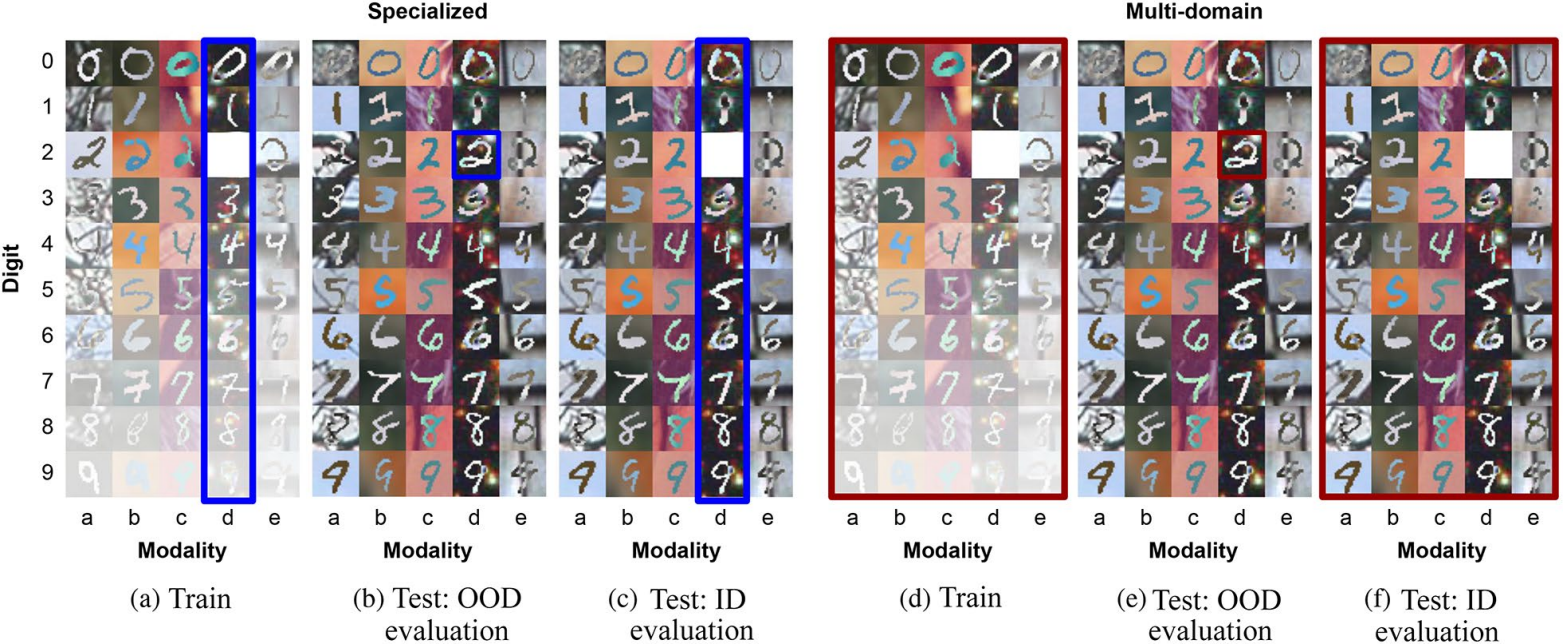
Training and evaluation procedures for specialized and multi-domain models: (**a**,**d**) represent the training data utilized by specialized and multi-domain models using $\mu _\text {digit}=0$, $\sigma _\text {digit}=5$, $\mu _\text {modality}=2$ and $\sigma _\text {modality}=3$ with a 100% out-of-distribution level for digit *2* and modality *d*. (**b**,**e**) show the out-of-distribution evaluation specifically for digit *2* and modality *d*. (**c**,**f**) demonstrate the in-distribution evaluation for all other digit/modality combinations except digit *2* and modality *d*.

In our experimental setup, we employ same datasets for both specialized and multi-domain models. The key distinction lies in the manner with which (part of) data the models are trained. To elaborate, for instance, in the case of PolyMNIST, we train and evaluate five distinct specialized models, each dedicated to classifying digits based on five modalities using the respective data from each modality. An example for modality-specific specialized models for the example data in Fig. 3(d) is shown in Fig. 4(a). In contrast, the multi-domain model leverages the entire available data for the same digit classification task, as shown in Fig. 4(d). It’s important to clarify that the choice between a multi-domain or specialized setup impacts the training and validation phases, determining which data partitions the models are exposed to. When it comes to the test phase, we assess each test image to predict its designated class. Therefore, the multi-domain doesn’t require the simultaneous input of all modalities during testing, e. g. in contrast to multi-modal learning.

## Experiments and results

### Experimental setup

#### Model architecture and hyperparameters

In our experiments, we utilize the pre-trained ResNet-18 architecture^48^, employing the cross-entropy loss and the AdamW optimizer^49^. PolyMNIST and ImageCLEFmedical training data is split into training and validation sets with a ratio of 0.75. For testing we use the validation data. For MedMNIST, we use the official train/validation/test splits. For MedMNIST and ImageCLEFmedical datasets, we evaluate and report the average of five random seeds. We train the models for 25 epochs and decay the learning rate by 0.1 every 5 epochs. For PolyMNIST, we set the learning rate to 0.005, use a batch size of 512 and employ a weight decay of 0.001. For MedMNIST, the learning rate is set to 0.001, batch size to 128, weight decay to 0.001. For ImageCLEFmedical, we use a learning rate of 0.0005, utilize a batch size of 128, set the weight decay to 0.00001. Note that, for hyperparameter tuning, we employed multi-domain models and conducted a grid search for optimizing learning rate, weight decay and different ResNet architectures such as ResNet-18, ResNet-34 and ResNet-50. We then repeated this for specialized models, with the hyperparameters largely aligned, ensuring that they did not affect the training convergence. The images in PolyMNIST and MedMNIST have the resolution of $$28\times 28$$$$\text {pixels}^2$$. We pre-process these images by resizing them to $$32\times 32$$$$\text {pixels}^2$$. As for ImageCLEFmedical, we center crop the images to ensure equal width and length, further augmenting them with a random 0x to 0.1x translation and resizing them to dimensions of $$224\times 224$$$$\text {pixels}^2$$.

#### Evaluation

We employ balanced accuracy as our evaluation metric, encompassing two distinct evaluation scenarios: out-of-distribution (OOD) accuracy and in-distribution (ID) accuracy. This involves evaluating the accuracy of each excluded combination and subsequently computing the average accuracy across all such combinations. For PolyMNIST, this approach results in a total of 50 evaluations (10 digits and 5 modalities), while for MedMNIST, we conduct 33 evaluations (11 organs and 3 views), and for ImageCLEFmedical, there are 54 evaluations (9 organs and 6 modalities) in total. We refer to the resulting metric as OOD average balanced accuracy. In addition, we calculate the average accuracy of all combinations except for the excluded ones and once again calculate the average of all the different combinations set. We designate this outcome as ID average balanced accuracy. All evaluation metrics are reported in test set. As an example for PolyMNIST, the difference between each of OOD and ID evaluation for both specialized and multi-domain models are shown in Fig. 4. Specifically, (a-c) illustrate the specialized setup, while (d-f) the multi-domain setup, featuring a 100% OOD level for digit *2* and modality *d*, ensuring that the models have no exposure to cases involving digit *2* and modality *d*. For the OOD evaluation, (b, e), both models are evaluated on test samples featuring digit *2* from modality *d*. For the ID evaluation, (c, f), the specialized model is assessed using test samples from modality *d* and all digits except *2*, while the multi-domain model undergoes evaluation for all other digit/modality combinations except digit *2* and modality *d*. Note that, in our work average balanced accuracy corresponds to the mean accuracy across all class/domain combinations, which is different from overall accuracy or balanced accuracy. Our evaluation approach involves a detailed breakdown based on class and domain.

### Results

#### PolyMNIST

We begin our analysis by assessing data diversity across various OOD levels. For this, we not only compare specialized and multi-domain models, but also introduce a modified version of specialized models, which we refer to as *specialized upsampled models*: we augment the data available to specialized models, ensuring that each digit is classified with an equal number of images for both the specialized upsampled and multi-domain models. The original PolyMNIST dataset provides a sufficient number of samples for this purpose. Thus, both specialized and multi-domain models have access to a maximum of 1000 images for each digit/modality combination, while the upsampled counterparts of the specialized models benefit from an expanded dataset, containing up to 5000 images for each such combination. Furthermore, we would like to mention that the scenario involving specialized upsampled models is not a realistic representation but is exclusively examined to assess the advantages and limitations associated with an augmented dataset.

**Fig. 5:**
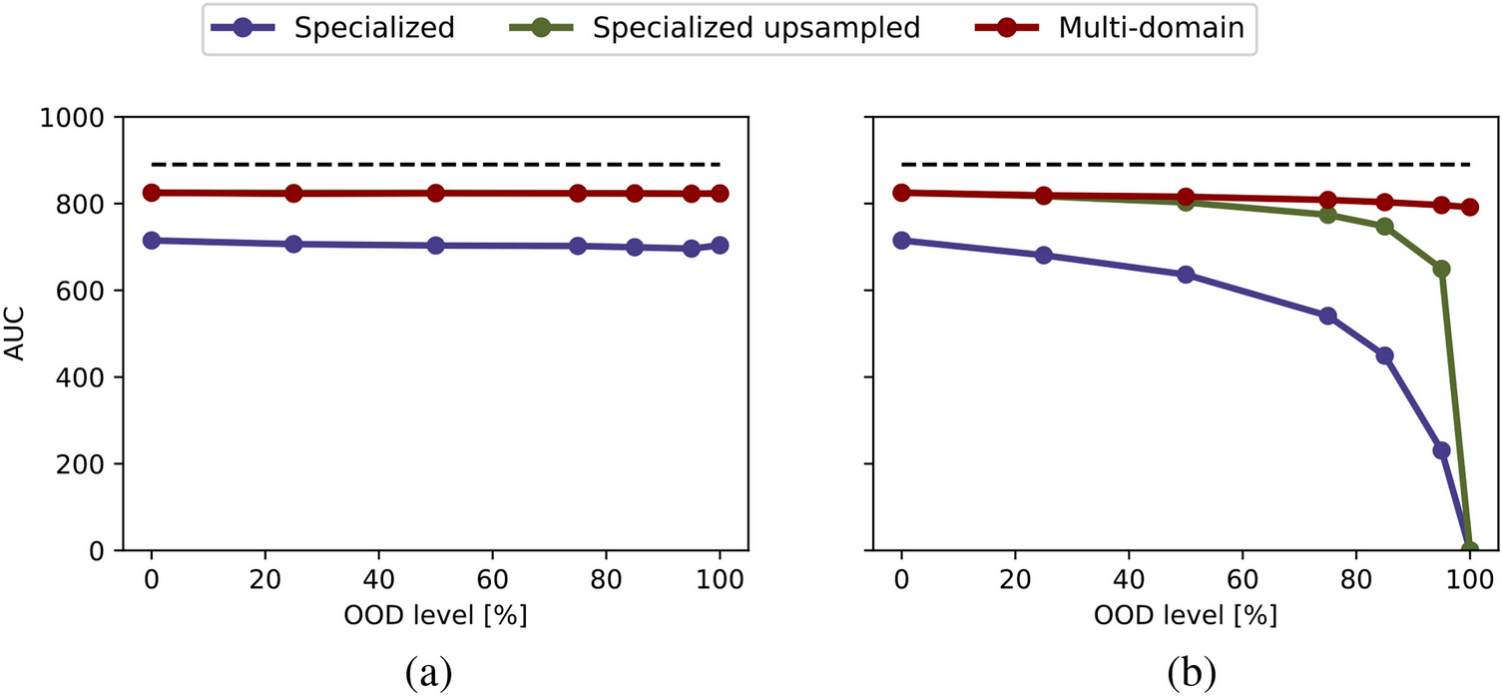
Evaluating out-of-distribution levels for PolyMNIST: (**a**) in-distribution and (**b**) out-of-distribution evaluation. Each point represents the area under the balanced accuracy curve (AUC) for the specialized (blue), specialized upsampled (green), and multi-domain (red) models across different evaluations of data distributions, with varying OOD levels shown on the x-axis.

Figure 5 compares the performance of specialized, specialized upsampled and multi-domain models. We compute the area under the curve (AUC) for both the OOD and ID average balanced accuracy curves across various data distributions. Each data point represents the AUC for different OOD level, where (a) provides a comparison of ID average balanced accuracy across different data distributions and OOD levels, while (b) shows OOD average balanced accuracy among specialized (blue), specialized upsampled (green), and multi-domain (red) models. Note that, in this experiment, the highest achievable AUC for a model is marked with a dashed black line. This is calculated using the highest possible average balanced accuracy, which can reach 1, and the total number of samples, maximum of 890 (as shown in Fig. A.1). Consequently, the dashed line reflects a maximum AUC of 890. The ideal outcome is represented by a flat line at maximum, indicating perfect performance unaffected by OOD levels.

**Fig. 6:**
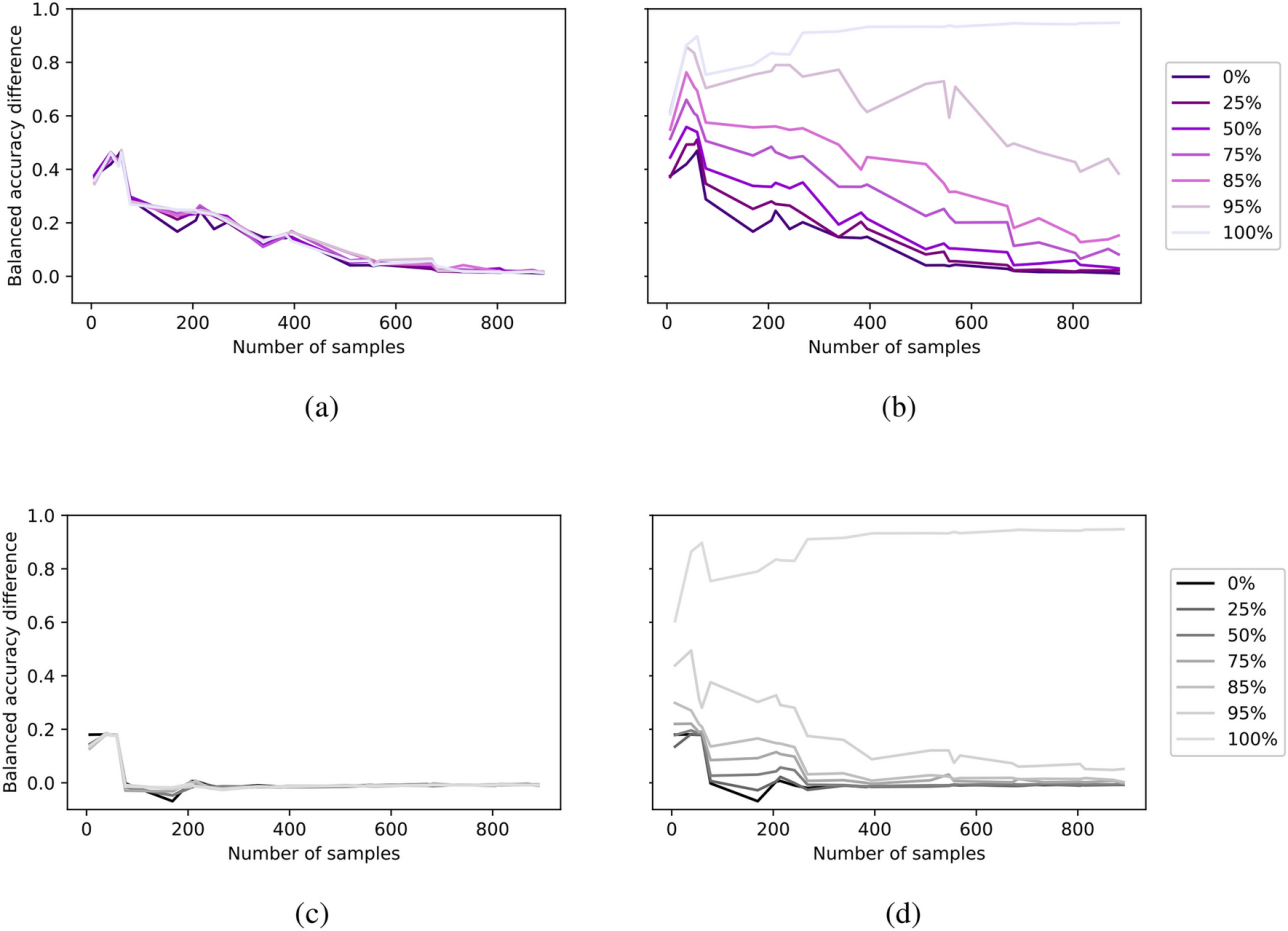
Evaluating amount of data for PolyMNIST: Comparison of balanced accuracy differences between specialized and multi-domain models (**a**,**b**) and between specialized upsampled and multi-domain models (**c**,**d**). Each line represents a different out-of-distribution level, illustrating how the accuracy differences between models vary across different data distribution scenarios, as shown on the x-axis.

Figure 6 provides an in-depth overview of the models across diverse data distributions. To facilitate a meaningful comparison across different distributions, we have organized the 24 distributions in ascending order based on their median values, as shown in Fig. A.1. The datapoints on x-axis represent these 24 different data distributions, labeled as the titles of each subfigure in Fig. A.1. They span from a minimum of 6 (median of the distribution in the first row, first column) to a maximum of 890 (median of the distribution in the second row, last column). We depict the balanced accuracy difference between specialized and multi-domain models (a,b) and specialized upsampled and multi-domain models (c,d). Each line represents OOD levels showing the differences between models during data distribution evaluations.

**Fig. 7:**
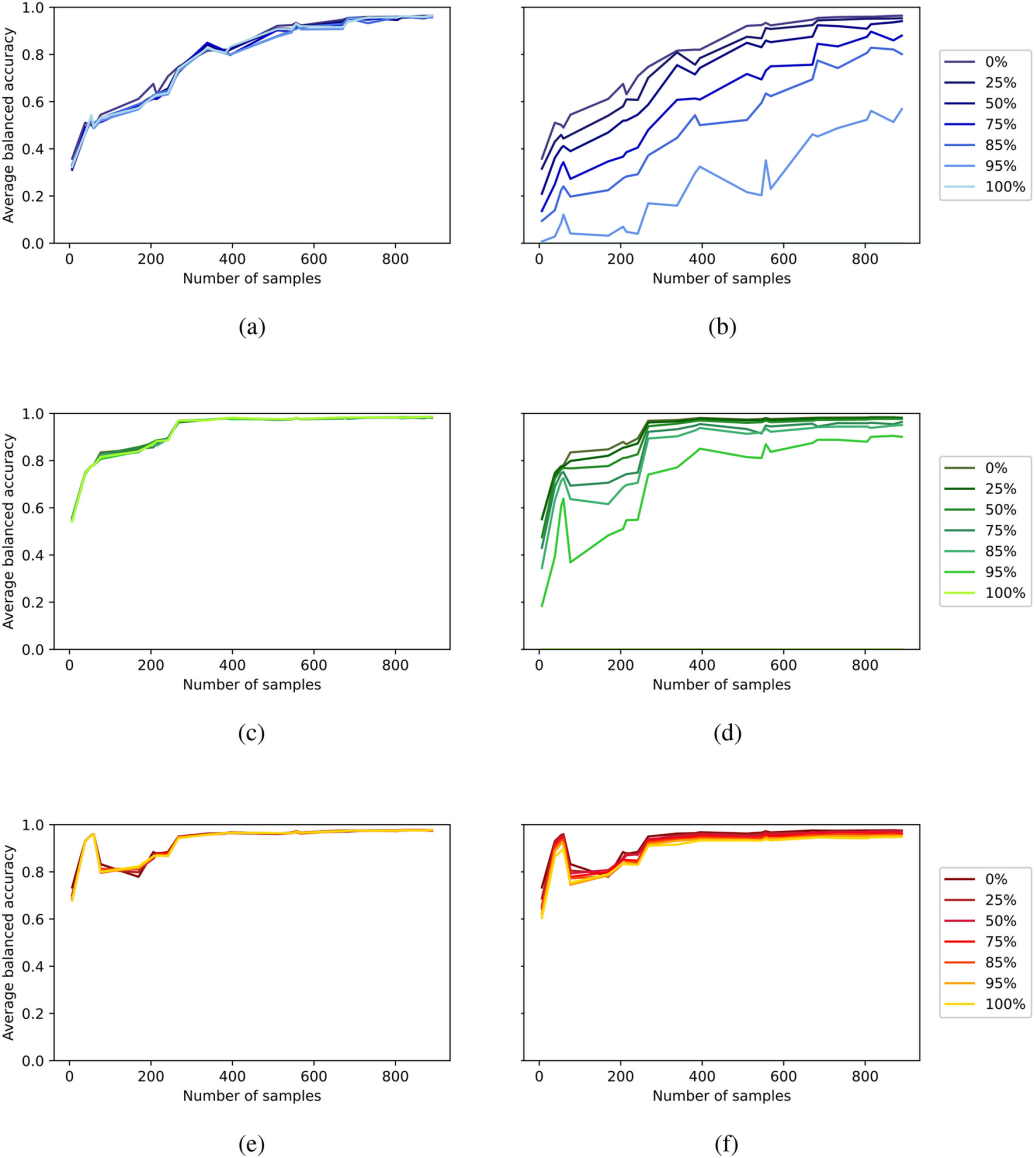
PolyMNIST performance across various data distributions: Average balanced accuracy is reported for specialized (**a**,**b**), specialized upsampled (**c**,**d**) and multi-domain (**e**,**f**) models. The x-axis represents different data distributions, while different color codes indicate different out-of-distribution levels. Results are shown for both in-distribution (**a**,**c**,**e**) and out-of-distribution (**b**,**d**,**f**) evaluations.

For a more detailed overview, please refer to Fig. A.7, which provides an overview of the average balanced accuracy scores for specialized (a,b), specialized upsampled (c,d) and multi-domain (e,f) models. These are presented across various distributions on the x-axis, and OOD levels, indicated by different color codes for both ID (a,c,e) and OOD (b,d,f) evaluation. Each data point in Fig. 7 represents the average accuracy obtained from 50 distinct experiments. In these experiments, one digit (out of 10 digits: 0 to 9) and one modality (out of 5 modalities: a to e) were systematically excluded from training and validation, and this process was repeated 50 times to encompass all possible class/modality combinations. Figure 5 was then calculated as the the AUC of each line representing the OOD levels in these figures. Specifically, in Fig. 5(a) and (b), the AUC values represented by the blue lines correspond to the areas under the curves for different OOD levels illustrated in Fig. 7(a) and (b). Similarly, for the red lines in Fig. 5(a) and (b), each calculated AUC value corresponds to the area under different OOD curves shown in Fig. 7(e) and (f). Additionally, we have computed the balanced accuracy difference for each OOD level in these figures, with multi-domain minus specialized models, and the outcomes are presented in Fig. 6.

When assessing the ID accuracy, our results indicate that varying levels of OOD scenarios do not notably impact ID accuracy. The performance of the specialized models are still lower than the specialized upsampled and multi-domain models, showing that ID accuracy can be compensated with higher number of samples. Upon analyzing OOD performance, a notable and consistent pattern emerges: as the OOD level increases, OOD accuracy declines noticeably for specialized and specialized upsampled models. Specialized models struggle to recover unseen (at OOD level 100%) or scarce encountered (at OOD level < 100%) digit/modality combinations, even when provided with larger sample sizes with specialized upsampled models. This stands in stark contrast to multi-domain models, which exhibit considerably greater resilience to this phenomenon. The difference becomes particularly pronounced for OOD levels >50%. This outcome can be attributed to the fact that the advantage of the multi-domain model comes not just from the quantity of data but from the diversity of data it encounters and the shared information across different modalities for the classification task. Specifically, we are referring to digit specific information as part of the shared information. Furthermore, across all models, we observe a consistent trend: both ID and OOD performance declines as the number of samples decreases. In Fig. 7(e) and (f), the accuracy does not exhibit a monotonic increase; instead, there is a peak at a smaller number of samples. We suspect that this peak at the small sample size is caused by the double descent phenomenon. This is because while the training error and loss function decrease in our experiments, the test error increases with a small number of samples.

In experiments, specialized models were trained exclusively with data from their corresponding modalities. For instance, a specialized model for modality *d* is exclusively trained using data from modality *d* and evaluated on modality *d*, as illustrated in Fig. 3(a-c). We further evaluated a scenario where the specialized model, initially trained on data from modality *d*, was tested on other modalities *a, b, c, e* to evaluate the robustness of the learned representations of the specialized models. For this we evaluated different OOD levels for digit *2* and modality *d*. In summary, training on one domain and testing on another leads to large drops of accuracy, showing that the specialized models have limited generalization capabilities to other domains. More specifically, for OOD level of 50%, the average balanced accuracy evaluated on digit *2* and domain *d* yielded 0.95, whereas on domains *a,b,c,e* yielded 0.71. Average balanced accuracy tested on all digits except digit *2* and on modality *d* yielded 0.94, in contrast, it was 0.77 for the modalities *a,b,c,e*. For OOD level of 85%, the average balanced accuracy evaluated on digit *2* and domain *d* yielded 0.87, whereas on domain *a,b,c,e* it was 0.59. Average balanced accuracy tested on all digits except digit *2* and on modality *d* was 0.94, in contrast it was 0.73 on domains *a,b,c,e*. In contrast, the multi-domain model learns a single model across all domains, is more robust to different domains, which results in OOD generalization, as shown in Figs. 6(b) and 7(f).

**Fig. 8:**
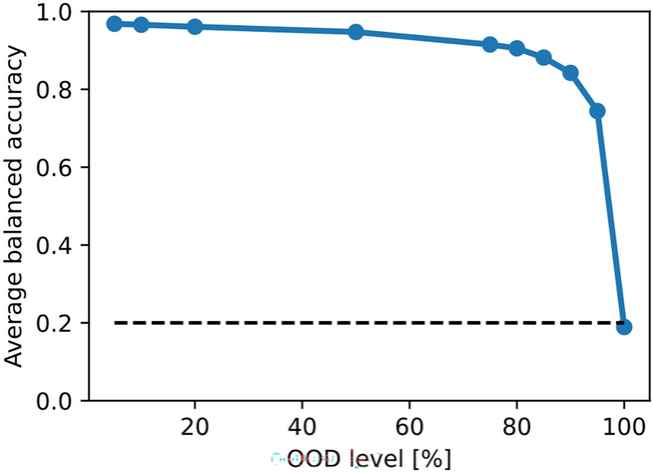
Control experiment for PolyMNIST using multi-domain model: We assess potential knowledge transfer and evaluate a scenario with limited information sharing. The dashed line represents the level of chance performance.

For testing potential knowledge transfer, we conducted another control experiment, where our goal was to evaluate a scenario where information sharing is constrained for multi-domain model. For this, we split the data from various modalities into two distinct domains, grouping classes as follows: 0 and 5 together as one class, 1 and 6 as another, and so on, with classes 4 and 9 comprising the final group. Thus, we split digits 0 through 4 into one domain and digits 5 through 9 into another. Consequently, our multi-domain model exploits both domains, with each class encompassing two dissimilar digits. We then run OOD level experiments for each digit and repeat this experiment for each of the original modalities *a* to *e*. In Fig. 8, we present the OOD evaluation results, showcasing the average balanced accuracy achieved by aggregating the experiments. The lower the OOD level, the higher is the accuracy. However, when the OOD level reaches 100%, the average accuracy declines to a level expected by chance. This observation shows the fact that at 100% OOD, there is no opportunity for knowledge transfer, emphasizing the absence of information sharing.

#### MedMNIST

**Fig. 9:**
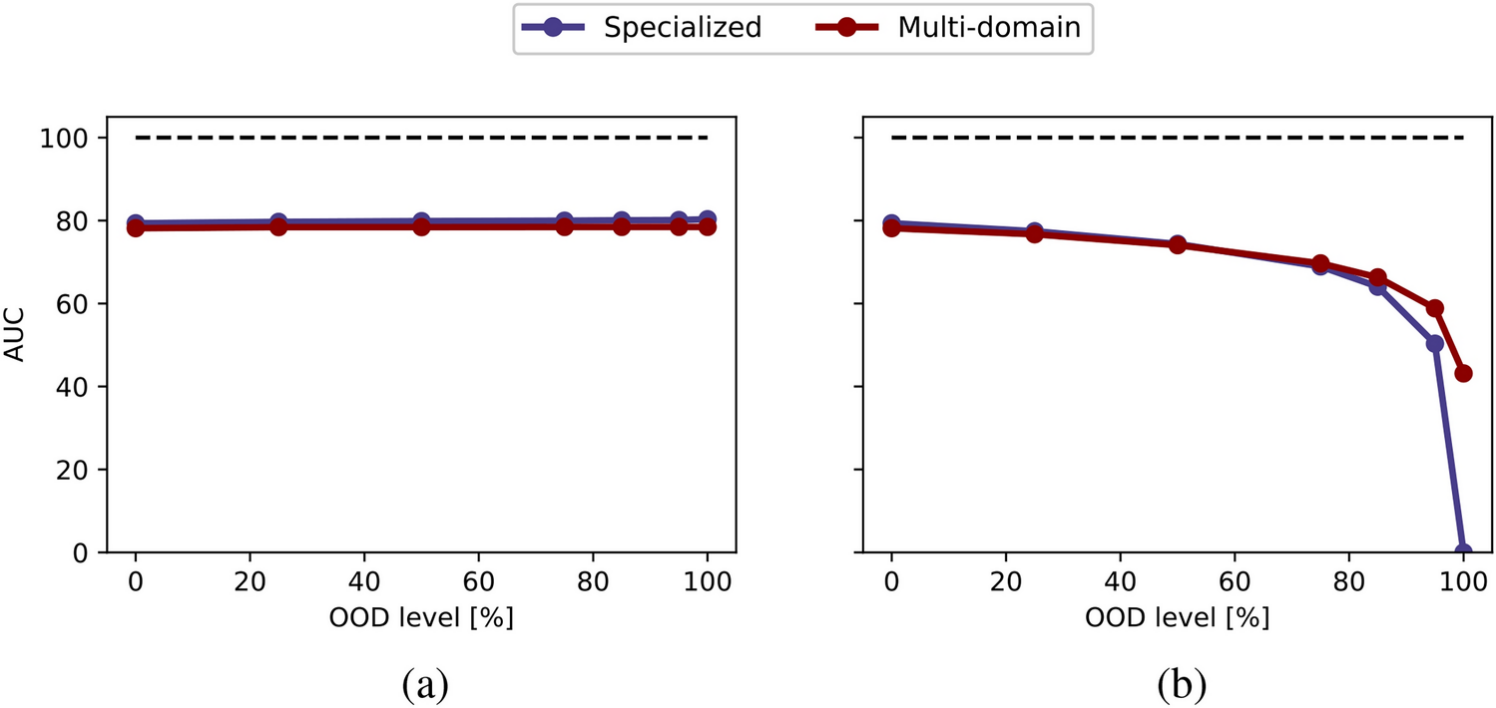
Evaluating out-of-distribution levels for MedMNIST: (**a**) in-distribution and (**b**) out-of-distribution evaluation. Each point represents the area under the balanced accuracy curve (AUC) across varying data availability (sampling percentage), with OOD levels shown on the x-axis.

**Fig. 10:**
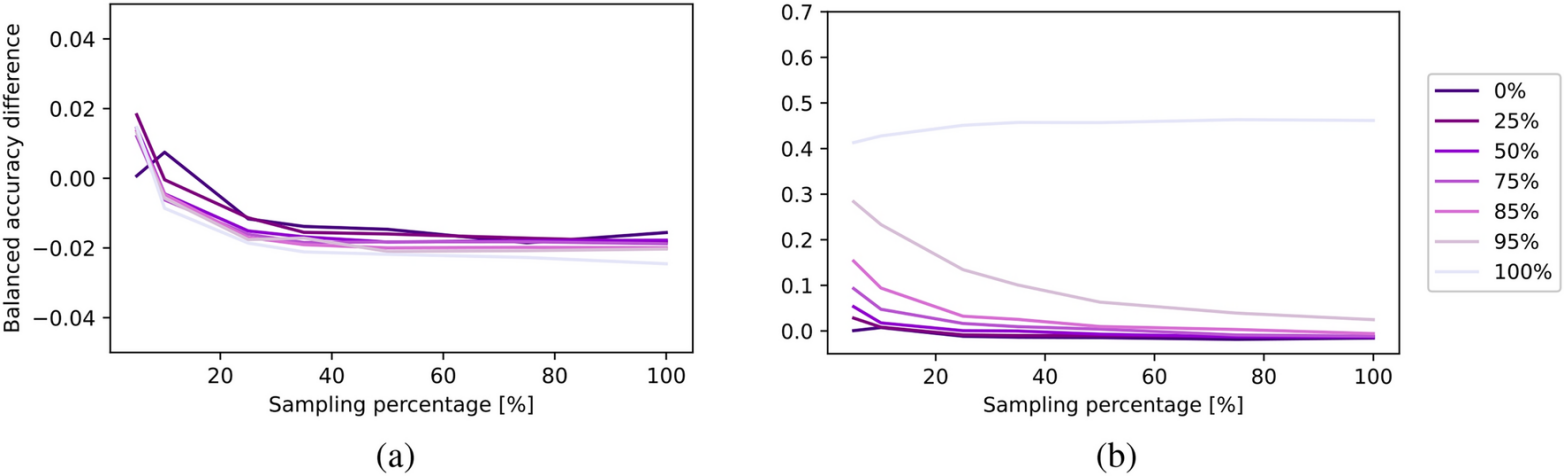
Evaluating amount of data for MedMNIST: (**a**) in-distribution and (**b**) out-of-distribution evaluation. Each line represents a different out-of-distribution level and shows the balanced accuracy difference between specialized and multi-domain models across varying amount of data (sampling percentage), as indicated on the x-axis.

In Fig. 9, we present a comparison of AUC values achieved by specialized and multi-domain models across various sampling percentages for both ID (a) and OOD (b) average balanced accuracy curves. The dashed black line, marked with a value of 100, represents the highest attainable AUC, which corresponds to a 100% sampling rate and perfect accuracy. Figure 10 provides a more comprehensive analysis of the specialized and multi-domain models with different amount of data for both ID evaluation (a) and OOD evaluation (b). Each line corresponds to different OOD levels and illustrates the differences between the models across different sampling percentages, as indicated on the x-axis. For a detailed overview, please refer to Fig. A.3, which presents an overview of the average balanced accuracy scores for specialized and multi-domain models. Furthermore, in Figs. A.4, A.5 and A.6, we report the AUC, accuracy differences, and model accuracies at view level.

When assessing the ID accuracy, our findings suggest that varying OOD levels do not significantly impact ID accuracy and both models exhibit similar levels of accuracy. For the OOD performance, as the OOD level increases, OOD accuracy experiences a noticeable decline for both models. This distinction between the models becomes particularly pronounced for OOD levels exceeding 75%. In the extreme case of a 100% OOD level, the specialized model’s accuracy drops, which makes it impossible for specialized models to predict fully unseen data. In contrast, the multi-domain model still benefits from shared information in this scenario, namely the organ specific information across views. Furthermore, across all models, both ID and OOD performance decrease as the number of samples for each digit/modality combination decreases.

**Fig. 11:**
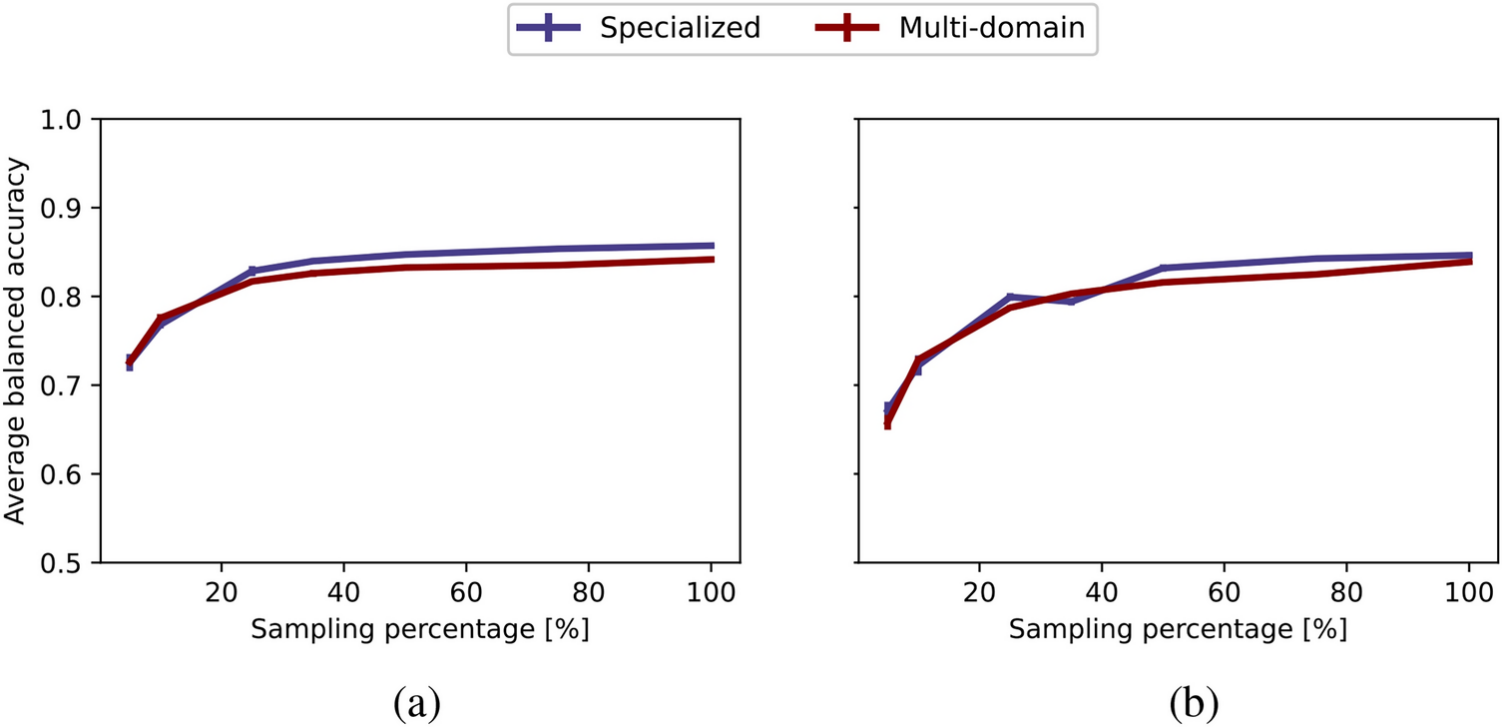
Evaluating data distribution in MedMNIST. In-distribution performance of specialized and multi-domain models across varying amount of data. Results are presented for two scenarios: (**a**) using the official data split and (**b**) applying a resampled uniform distribution.

Figure 2(a) displays the image distribution across organ/view combinations in MedMNIST’s train/validation/test splits. Across all splits, the axial view consistently contains the highest number of images, notably with the liver in the axial view having the most images in both the train and test splits. To investigate whether the matching ID accuracy between specialized and multi-domain models can be attributed to data distribution, we conducted a control experiment. For this, we restructured the data by resampling so that each organ/view combination contained 600 samples for training and 95 samples for the validation split, aligning with the minimum sample size observed in the official training and validation split. We repeat the experiment with using the resampled dataset and for different amount of data using sampling percentage. As an example, with a 50% sampling percentage, each organ/view combination benefits from 300 training and 48 validation samples. Figure 11 provides a comparison of these different distributions for OOD level of 0%. These show that the data distribution has a negligible impact on ID accuracy, as the resampled uniform distribution data continues to demonstrate matching ID accuracy between specialized and multi-domain models.

#### ImageCLEFmedical

**Fig. 12:**
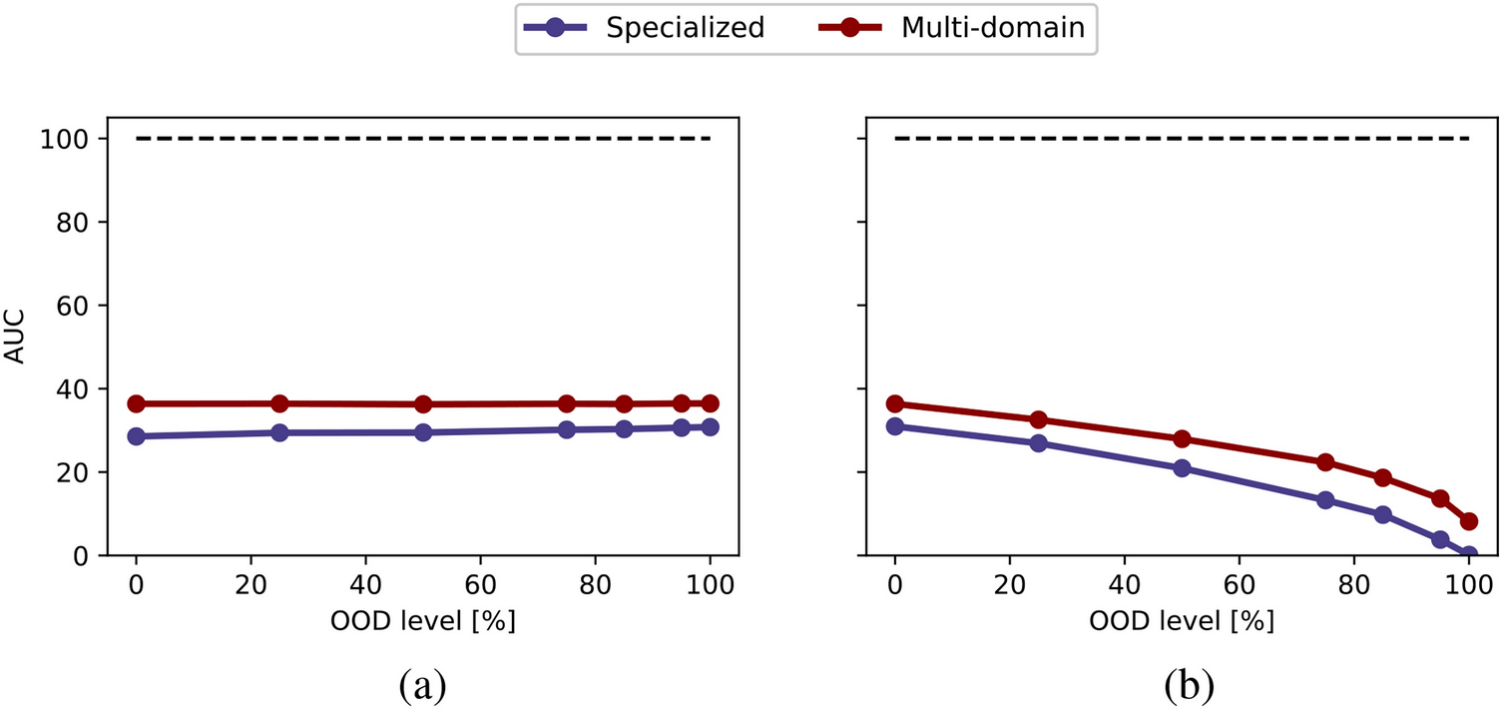
Evaluating out-of-distributionlevels for ImageCLEFmedical: (**a**) in-distribution and (**b**) out-of-distribution evaluation. Each point represents the area under the balanced accuracy curve (AUC) across varying data availability (sampling percentage), with varying OOD levels shown on the x-axis.

**Fig. 13:**
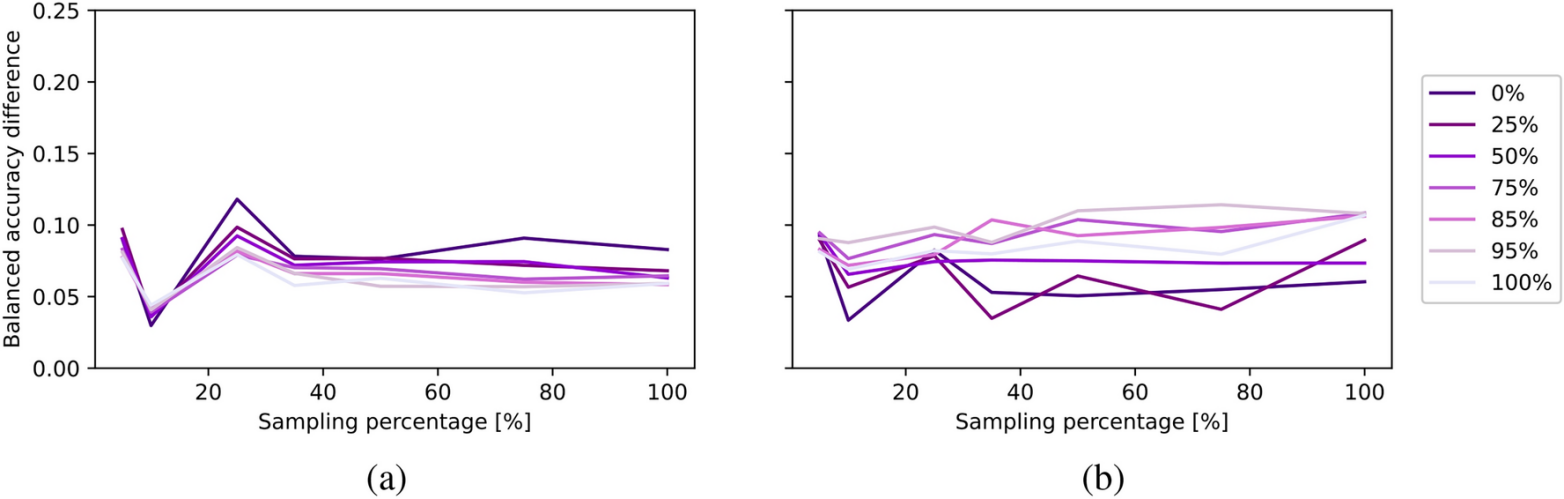
Evaluating amount of data for ImageCLEFmedical: (**a**) in-distribution and (**b**) out-of-distribution evaluation. Each line represents a different out-of-distribution level and shows the balanced accuracy difference between specialized and multi-domain models across varying amount of data (sampling percentage), as indicated on the x-axis.

In Fig. 12, we compare the models in terms of their AUC values under ID (a) and OOD (b) average balanced accuracy curves. Figure 13 presents a more in-depth comparison of the specialized and multi-domain models in terms of varying amount of data for both ID (a) and OOD (b) evaluation. For a detailed overview, please refer to Fig. A.7, which provides a summary of the average balanced accuracy scores for specialized and multi-domain models.

For ID accuracy, our findings are consistent with the outcomes observed in PolyMNIST and MedMNIST studies. Here, variations in OOD scenarios do not significantly impact ID accuracy. In contrast to MedMNIST, the multi-domain model demonstrates an 8% improvement in accuracy, mirroring the findings in PolyMNIST where multi-domain model outperformed specialized models. This result is particularly intriguing given that the utilized images are unprocessed and have remarkable diversity. Regarding OOD performance, as the OOD level increases, both models experience a noticeable decline. Importantly, the multi-domain model maintains a consistent 8% advantage across all OOD levels, where the multi-domain model continues to benefit from shared information for both ID and OOD evaluation, even in data-limited and OOD scenarios.

Comparing Fig. 5(b) with Figs. 9(b) and 12(b), the multi-domain approach appears to be less robust to OOD scenarios in medical settings compared to the toy dataset PolyMNIST experiments. This may be because, in PolyMNIST, the shared information-the digit-is more obvious, clearly showcasing the advantage of the multi-domain model. In contrast, the shared information in medical datasets is less immediately apparent, making the models for medical datasets less robust. Nevertheless, the multi-domain model still demonstrates a clear advantage by effectively leveraging cross-modality information for higher OOD levels.

## Discussion and conclusion

Motivated by the recent advancements in foundation models exploiting diverse data sources and demonstrating exceptional generalization abilities, this work introduced a multi-domain strategy and compared its performance with the single domain specialized approach, particularly within the context of medical image analysis, where the latter is the norm. These are evaluated in scenarios involving OOD and data-limited scenarios using three datasets, such as the toy dataset PolyMNIST^4^, as well as two medical datasets, MedMNIST^5^ and ImageCLEFmedical^6^ and obtain following key conclusions:

- Multi-domain models outperform specialized models in OOD and data-limited scenarios, capitalizing on their ability to leverage shared information across diverse domains.
- Multi-domain models consistently either match or excel specialized models in terms of their ID accuracy.
- Specialized models can compensate for ID accuracy with a higher number of samples. However, they face considerable challenges in recovering OOD accuracy for classes that are entirely unseen or encountered only infrequently, even when provided with larger sample sizes.
- The level of OOD scenario does not impact ID accuracy for any of the models, indicating the robustness in preserving ID accuracy across varying OOD levels.

Based on our findings, the advantage of multi-domain model is particularly pronounced for OOD evaluation when OOD level exceeds 80%. An OOD level surpassing 80% highlights instances where the model encounters class/domain combinations either extremely rarely or never before. Such scenarios are common in medical applications, particularly those involving rare diseases or conditions. Accessing medical conditions from diverse data domains could be beneficial, particularly when a specific condition hasn’t been frequently observed within a particular domain in the training data. It’s worth noting that the extent of the advantage of knowledge transfer between domains is limited upon the availability of shared information.

While this work focuses on the classification task, we recognize the importance of investigating the applicability of the multi-domain approach to more complex tasks. Future research could explore how the multi-domain approach might be adapted or extended to these more complex tasks, such as image segmentation or reconstruction, potentially offering new insights into its broader utility for medical image analysis. As a future direction, understanding the underlying mechanisms behind the generalization capabilities of multi-domain models for OOD and data-limited scenarios is a crucial direction. Deeper investigations into the information sharing within these models hold the potential to yield more efficient strategies for knowledge transfer and domain adaptation. Moreover, a potential future work is to improve multi-domain models by incorporating domain-specific knowledge into the training process^50–53^, e.g. through the use of alternative loss functions. Furthermore, addressing the scalability of these models for real-world, large-scale applications remains a pressing concern for medical image analysis. Future research can concentrate on working with refining these models for efficiency and ensuring their practicality in real-world resource-constrained environments. Additionally, the exploration of more diverse datasets and problem domains will be essential for validating and extending our findings.

In summary, our work underlines the effectiveness of multi-domain models in tackling OOD and data-limited challenges, offering promising avenues for their application in medical image analysis where such challenges are prevalent. These insights contribute to the ongoing exploration and implementation of large scale models in diverse fields and applications.

## Supplementary Information

Supplementary Information.

## Data Availability

We used publicly available datasets in this work. The code can be found in a GitHub repository at https://github.com/eceoezkan/multi_domain_medical.
