# Supplementary material for: Multi-domain improves classification in out-of-distribution and data-limited scenarios for medical image analysis
Source: Sci Rep. 2024 Oct 18;14:24412. doi: 10.1038/s41598-024-73561-y (PMC11487066; doi:10.1038/s41598-024-73561-y)
Supplement: Supplementary file 1 — Supplementary Information. [file 41598_2024_73561_MOESM1_ESM.pdf]

A Supplementary Material

A.1 PolyMNIST

In Figure A.1, we present 24 distinct data distributions, each representing the number of samples within training and validation splits. To be able to summarize and visualize these, we calculated the median of each of the 24 distributions. These values are displayed in the titles of each subfigure in Figure A.1 and correspond to the x-axis values for the evaluations in Figure 6 and Figure 7.

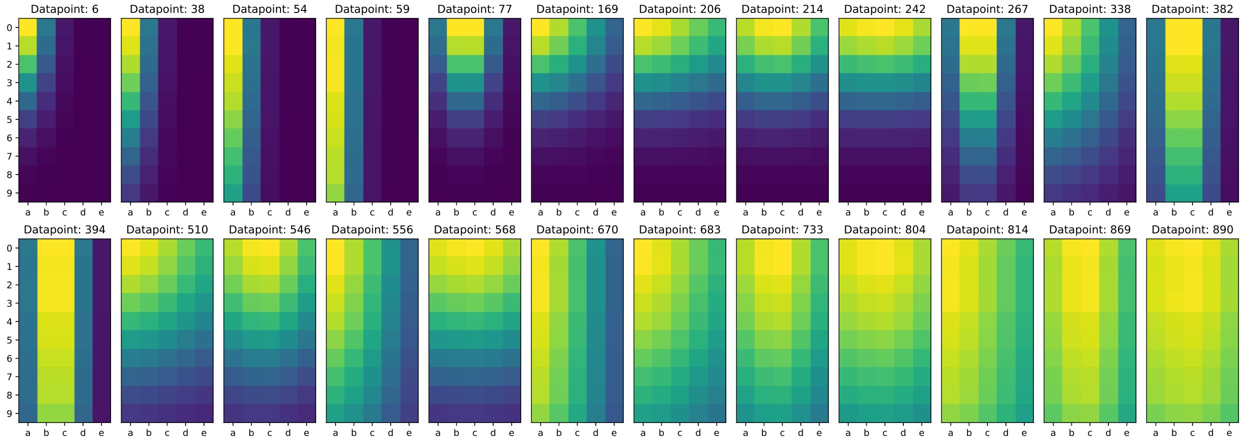

Figure A.1. 24 distinct data distributions, each representing the number of samples within various training and validation splits. These are characterized by diverse probability distributions for digit and modality combinations. They are organized in ascending order, based on their respective median values.

We conducted an additional experiment, mirroring the amount of data experiments conducted with MedMNIST and ImageCLEFmedical datasets using the sampling percentage. For this, we used a uniform sample distribution for each digit/modality combination having 1000 samples. Subsequently, we performed sampling at rates of $\{5, 10, 25, 35, 50, 75, 100\}\%$, resulting further in a uniform distribution. For example, when using a 10% sampling percentage, we obtained 100 samples for each digit/modality combination. Figure A.2 reports AUC under the average balanced accuracy curves across sampling percentage for various OOD levels for PolyMNIST. Notably, these results underscores the similar trend to those observed in Figure 5.

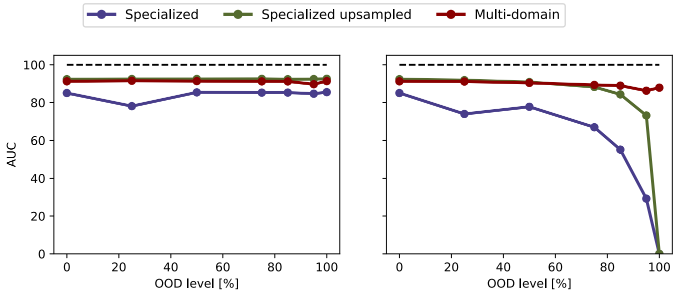

(a) PolyMNIST: In-distribution (b) PolyMNIST: Out-of-distribution

Figure A.2. Evaluating out-of-distribution levels for PolyMNIST: (a) in-distribution and (b) out-of-distribution evaluation. Each point shows the area under the balanced accuracy curve (AUC) for the specialized (blue), specialized upsampled (green), and multi-domain (red) models across varying sampling percentages, with OOD levels shown on the x-axis.

A.2 MedMNIST

Table A.1 displays the distribution of images in the MedMNIST dataset categorized by organs including *bladder, left femoral head, right femoral head, heart, left kidney, right kidney, liver, left lung, right lung, pancreas* and *spleen*, as well as by *axial, coronal*, and *sagittal* views.

	(i) Train			(ii) Validation			(iii) Test		
**Organ**	**Axial**	**Coronal**	**Sagittal**	**Axial**	**Coronal**	**Sagittal**	**Axial**	**Coronal**	**Sagittal**
Bladder	1956	1153	1148	321	191	188	1036	833	811
Left femoral head	1408	626	637	233	102	104	784	442	439
Right femoral head	1359	608	615	225	96	95	793	441	447
Heart	1474	600	721	392	202	246	785	421	510
Left kidney	3963	1088	1132	568	132	140	2064	732	704
Right kidney	3817	1170	1119	637	157	159	1965	737	693
Liver	6164	2986	3464	1033	429	491	3285	1836	2078
Left lung	3919	1002	741	1033	347	261	1747	550	397
Right lung	3929	1022	803	1009	352	275	1813	558	439
Pancreas	3031	1173	2004	529	179	280	1622	750	1343
Spleen	3561	1572	1556	511	205	213	1884	968	968

Table A.1. Number of images for MedMNIST dataset for (i) training, (ii) validation, and (iii) test set.

Figure A.3 presents a comprehensive summary of the average balanced accuracy scores for both specialized and multi-domain models for different OOD levels and amount of data. We report the mean and standard deviation (as the error bar) of the test accuracy across five random seeds.

Figure A.4 reports the AUC, Figure A.5 highlights the accuracy differences, and Figure A.6 shows the accuracy of the specialized and multi-domain models at a more granular view level.

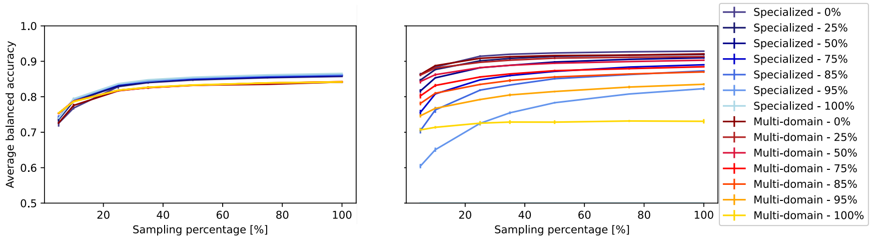

(a) MedMNIST: In-distribution

(b) MedMNIST: Out-of-distribution

Figure A.3. Comparison of models across varying amounts of data for MedMNIST: Average balanced accuracy is reported for specialized and multi-domain models across different sampling rates, shown on the x-axis, and out-of-distribution levels, indicated by different color codes. Results are presented for both in-distribution (a) and out-of-distribution (b) evaluations. The mean and standard deviation of the test accuracy are reported across five random seeds.

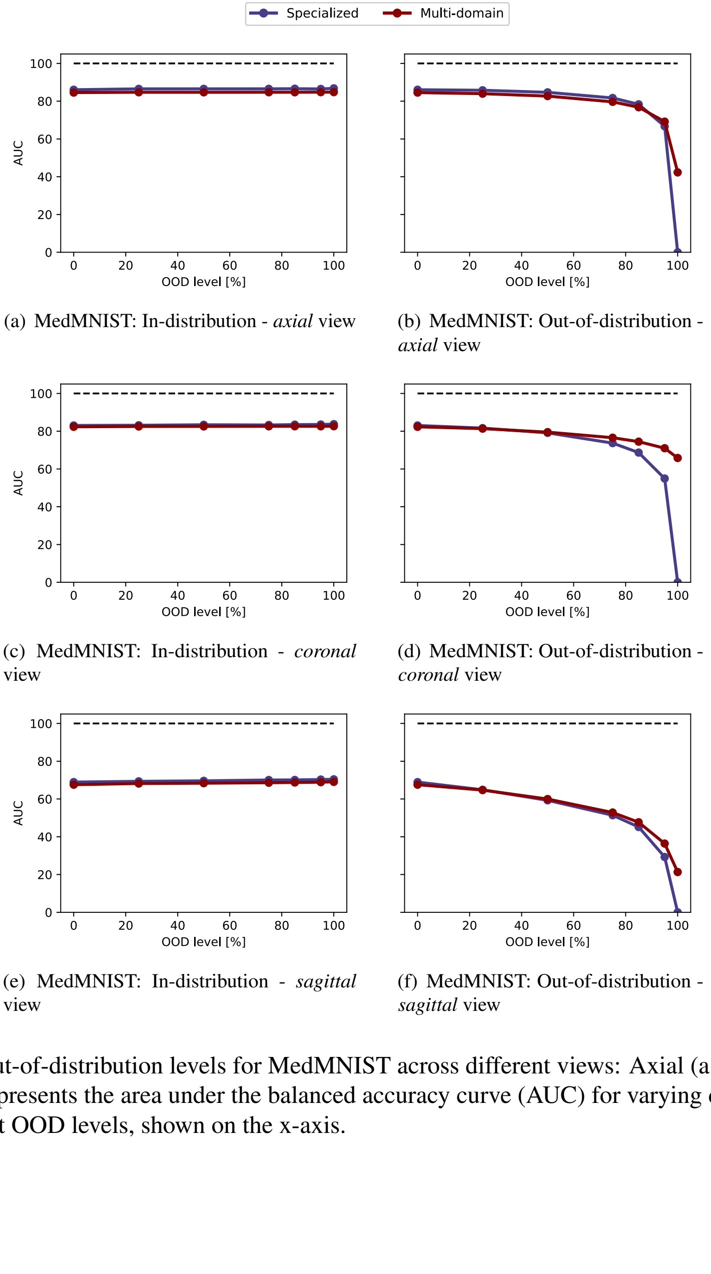

(a) MedMNIST: In-distribution - *axial* view

(b) MedMNIST: Out-of-distribution - *axial* view

(c) MedMNIST: In-distribution - *coronal* view

(d) MedMNIST: Out-of-distribution - *coronal* view

(e) MedMNIST: In-distribution - *sagittal* view

(f) MedMNIST: Out-of-distribution - *sagittal* view

Figure A.4. Evaluating out-of-distribution levels for MedMNIST across different views: Axial (a,b), coronal (c,d), and sagittal (e,f). Each point represents the area under the balanced accuracy curve (AUC) for varying data availability (sampling percentage) across different OOD levels, shown on the x-axis.

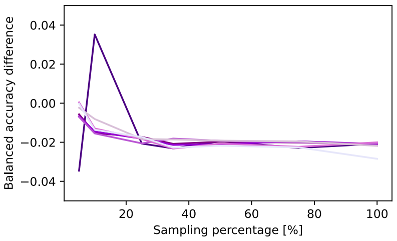

(a) MedMNIST: In-distribution - *axial* view

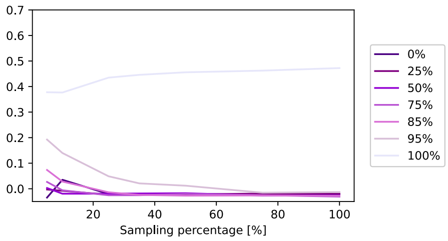

(b) MedMNIST: Out-of-distribution - *axial* view

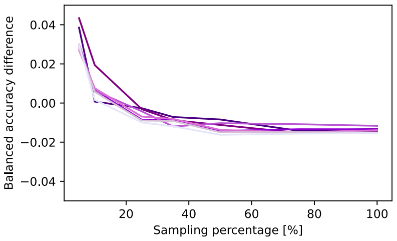

(c) MedMNIST: In-distribution - *coronal* view

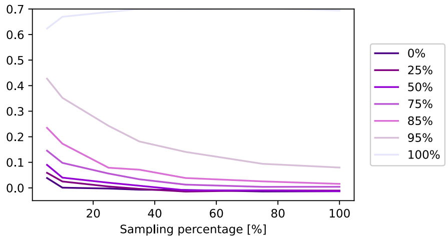

(d) MedMNIST: Out-of-distribution - *coronal* view

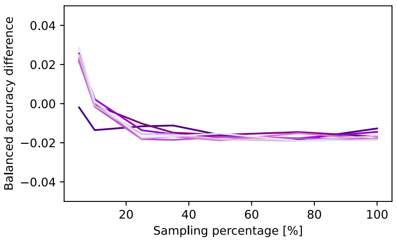

(e) MedMNIST: In-distribution - *sagittal* view

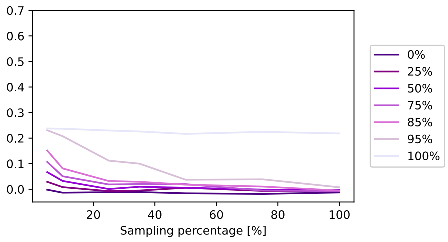

(f) MedMNIST: Out-of-distribution - *sagittal* view

Figure A.5. Evaluating amount of data for MedMNIST across different views: axial (a,b), coronal (c,d), and sagittal (e,f). Each line represents a different out-of-distribution level and presents the balanced accuracy difference between specialized and multi-domain models across varying amount of data (sampling percentage), as indicated on the x-axis.

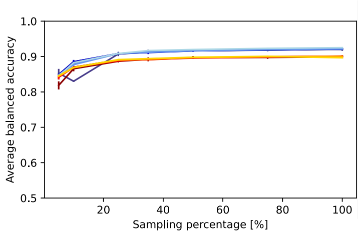

(a) MedMNIST: In-distribution - *axial* view

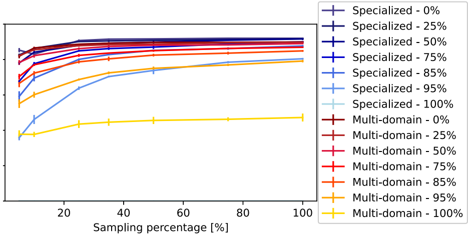

(b) MedMNIST: Out-of-distribution - *axial* view

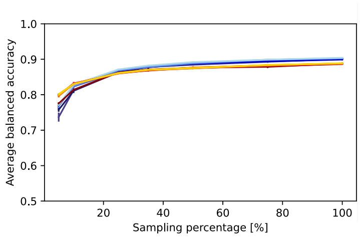

(c) MedMNIST: In-distribution - *coronal* view

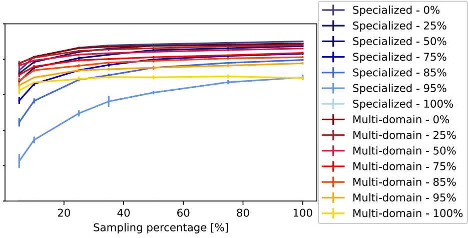

(d) MedMNIST: Out-of-distribution - *coronal* view

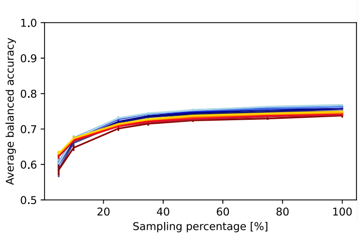

(e) MedMNIST: In-distribution - *sagittal* view

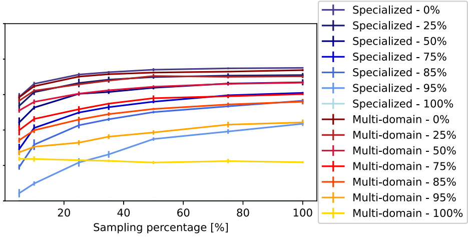

(f) MedMNIST: Out-of-distribution - *sagittal* view

Figure A.6. Comparison of models across various amount of data for MedMNIST, evaluated for each of the views: axial (a,b), coronal (c,d), and sagittal (e,f). Average balanced accuracy is reported for specialized and multi-domain models across different sampling rates, shown on the x-axis, and out-of-distribution levels, indicated by different color codes. Results are presented for both in-distribution (a,c,e) and out-of-distribution (b,d,f) evaluations, with mean and standard deviation of the test accuracy reported across five random seeds.

A.3 ImageCLEFmedical

Table A.2 displays the distribution of images in the ImageCLEFmedical dataset categorized by organs including *pelvis, vertebral column, lung, urinary bladder, right ventricular structure, stomach, pulmonary artery structure, anterior descending branch of the left coronary artery*, and *left kidney*, as well as imaging modalities *CT, X-ray, MRI, US, AG*, and *PET*.

(i) Train + Validation

Organ	CT	X-ray	MRI	US	AG	PET
Pelvis	826	1747	262	45	20	23
Vertebral column	23	1134	101	8	3	2
Lung	492	313	14	63	6	24
Urinary bladder	251	116	116	149	14	10
Right ventricular structure	70	36	59	507	28	0
Stomach	311	171	39	71	21	3
Pulmonary artery structure	219	42	24	109	74	0
Ant. desc. b. left cor. artery	35	7	23	20	373	0
Left kidney	275	29	51	64	8	2

(ii) Test

Organ	CT	X-ray	MRI	US	AG	PET
Pelvis	143	45	22	2	2	0
Vertebral column	4	7	7	2	1	1
Lung	79	53	0	11	2	3
Urinary bladder	37	11	6	19	2	0
Right ventricular structure	10	4	3	47	0	0
Stomach	19	15	3	4	1	0
Pulmonary artery structure	24	2	3	8	4	0
Ant. desc. b. left cor. artery	5	0	3	2	39	0
Left kidney	23	1	3	5	1	0

Table A.2. Number of images for ImageCLEFmedical dataset for (i) train and validation and (ii) test set.

Figure A.7 presents a comprehensive summary of the average balanced accuracy scores for both specialized and multi-domain models for different OOD levels and amount of data. We report the mean and standard deviation (as the error bar) of the test accuracy across five random seeds.

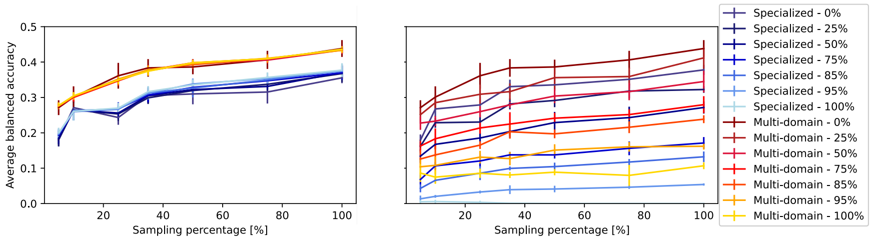

(a) ImageCLEFmedical: In-distribution

(b) ImageCLEFmedical: Out-of-distribution

Figure A.7. Comparison of models across various amount of data for ImageCLEFmedical. Average balanced accuracy is reported for specialized and multi-domain models across different sampling rates, shown on the x-axis, and out-of-distribution levels, indicated by different color codes. Results are presented for both in-distribution (a) and out-of-distribution (b) evaluations, with mean and standard deviation of the test accuracy reported across five random seeds.
